# Exposure to Glycolytic Carbon Sources Reveals a Novel Layer of Regulation for the MalT Regulon

**DOI:** 10.1155/2011/107023

**Published:** 2011-08-14

**Authors:** Sylvia A. Reimann, Alan J. Wolfe

**Affiliations:** Department of Microbiology and Immunology, Stritch School of Medicine, Loyola University Chicago, 2160 S. First Avenue, Building 105, Maywood, IL 60153, USA

## Abstract

Bacteria adapt to changing environments by means of tightly coordinated regulatory circuits. The use of synthetic lethality, a genetic phenomenon in which the combination of two nonlethal mutations causes cell death, facilitates identification and study of such circuitry. In this study, we show that the *E*. *coli ompR malT* ^con^ double mutant exhibits a synthetic lethal phenotype that is environmentally conditional. MalT^con^, the constitutively active form of the maltose system regulator MalT, causes elevated expression of the outer membrane porin LamB, which leads to death in the absence of the osmoregulator OmpR. However, the presence and metabolism of glycolytic carbon sources, such as sorbitol, promotes viability and unveils a novel layer of regulation within the complex circuitry that controls maltose transport and metabolism.

## 1. Introduction

Synthetic lethality, a phenomenon in which the combination of two nonlethal mutations causes death, is a powerful genetic tool that can, in an unbiased fashion, identify novel connections between cellular processes that function together to permit survival in a stressful environment. However, because the double mutant dies, investigating the process by which death occurs can be difficult. If, however, some permissive condition exists that permits survival of the double mutant, then the study of the death process is greatly facilitated, because genetic manipulations can be performed under permissive conditions and the consequences of those manipulations studied at nonpermissive conditions. Here, we explore one environmental condition (exposure to glycolytic carbon sources) that permits survival of the previously reported synthetic lethal mutant *ompR malT* ^con^ [1], which lacks the response regulator OmpR whilst harboring a constitutively active MalT^con^ protein. 

As osmolality increases, the two-component response regulator OmpR becomes activated by the receipt of a phosphoryl group from its cognate sensor kinase EnvZ [2, 3]. Upon phosphorylation, OmpR controls more than 100 genes associated with outer membrane biogenesis, osmoregulation, and envelope stress [4, 5].

**Figure 1:**
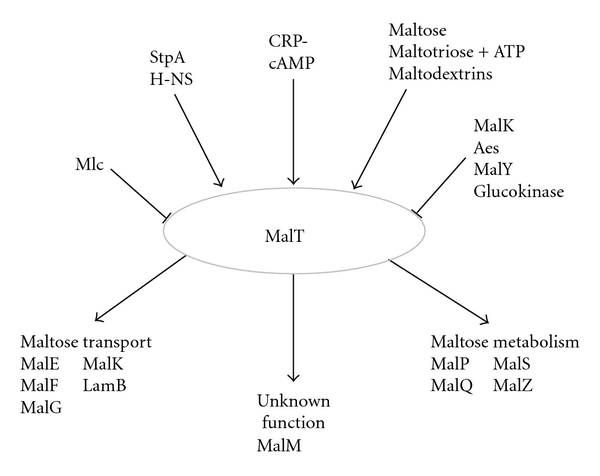
Regulation of MalT and its regulon.* malT* transcription is activated by the global regulator CRP-cAMP and repressed by Mlc. Translation of MalT is activated by H-NS and StpA. Activation of MalT activity can be attained by the binding of maltotriose, whereas it is inhibited by interaction with MalK, MalY, Aes, or glucokinase. Upon activation, MalT positively regulates expression of proteins that facilitate maltose uptake and metabolism.

MalT is the central regulator of all *mal* genes [6, 7] (Figure 1). The* mal* genes encode proteins involved in transport and metabolism of maltose and maltodextrins. The outer membrane porin LamB facilitates the uptake of maltose and maltodextrins into the periplasm, where these sugars are bound by the maltose-binding protein MalE and delivered to the MalFGK_2_ transporter [8]. Following transport into the cytoplasm, the sugars are metabolized [7]. MalT itself is activated by the maltose metabolite maltotriose and inhibited by MalK, MalY, Aes, and glucokinase [9–12]. The nucleoid proteins H-NS and StpA positively regulate MalT translation [13, 14]. *malT* transcription is activated by the cAMP-CRP complex, which renders it subject to catabolite repression [15, 16]. Finally, Mlc represses *malT *transcription [17].

Under non-permissive conditions, the *ompR malT* ^con^ mutant displays a set of striking phenotypes. Colonies on plates are translucent and form papillae [1]. In liquid medium, the culture loses turbidity upon entry into late exponential phase [1], because the inner membrane disintegrates [18]. The main cause for these phenotypes is elevated LamB expression in the absence of OmpR: deletion of *lamB *permits survival [1], while genetic suppressors that reduce LamB expression also permit survival [18]. Similar to genetic suppressors, any environmental condition that reduces LamB levels should permit cell survival. For example, the *ompR malT* ^con^ mutant survives on minimal medium supplemented with glucose as the sole carbon source [1], almost certainly, because glucose causes catabolite repression of transcription from the *malT*and *malK *promoters and thus reduced expression of LamB [15, 19].

In this study, we identified an additional permissive environmental condition that supports survival of the *ompR malT* ^con^ mutant: the presence of noncatabolite repressing glycolytic carbon sources in the growth medium. Characterization of this condition allowed us to unveil a new regulatory layer of the MalT regulon. We hypothesize that this regulation requires metabolism of glycolysis-associated carbon sources.

## 2. Materials and Methods

### 2.1. Bacterial Strains, Bacteriophage, Transcriptional Fusions, and Plasmids

All bacterial strains used in this study are listed in Table 1. All strains evaluated were derivatives of *E. coli* AJW678 [20]. Derivatives were constructed by generalized transduction with P1*vir*, as described [21]. 

The transcription fusion *malE*pΔ92-*lac* was a generous gift from Winfried Boos (Universität Konstanz, Germany) and was described previously [11].

The *malT* ^con^ allele (*malTc-1)* used in this study was a generous gift from Linda Kenney (University of Illinois at Chicago, IL, USA). It harbors a T949A base substitution and encodes the MalT^con^W317R protein. Unless otherwise mentioned, deletion alleles were derived from the Keio collection [22].

### 2.2. Media and Growth Conditions

Because the *ompR malT* ^con^ mutant is conditionally lethal, cells were grown overnight under a permissive condition: 22°C in M63 minimal salts [21] with 22 mM sorbitol as the sole carbon source and supplemented with 100 *μ*g mL^−1^ L-threonine, L-histidine, L-leucine, L-methionine, L-tryptophan, and 10 *μ*g thiamine ml^−1^. Whenever required, kanamycin (40 *μ*g ml^−1^), chloramphenicol (25 *μ*g ml^−1^), ampicillin (100 *μ*g ml^−1^), or tetracycline (15 *μ*g ml^−1^) was added. 

For tests of lethality, an inoculum from the overnight culture was subcultured at 37°C in LB (1% (w/v) tryptone, 0.5% (w/v) yeast extract, 0.5% (w/v) NaCl). LB agar plates also contained 1.5% (w/v) bacto agar. These growth conditions were considered non-permissive. Whenever required, carbon sources were added at a concentration of 22 mM. Cell growth was monitored spectrophotometrically (Beckman Instruments DU640) at 600 nm (OD_600_).

### 2.3. Promoter Activity Assays

To monitor *malE*pΔ92-*lac* promoter activity, cells were grown aerobically with 250 rpm agitation at 37°C. At various time points during growth, 50 *μ*L aliquots were harvested and added to 50 *μ*L All-in-One *β*-galactosidase reagent (Pierce Biotechnology). *β*-galactosidase activity was determined quantitatively in a microtiter format, as described previously [23]. To avoid misleading results caused by lysing cells that spill *β*-galactosidase into the growth medium, we only considered *β*-galactosidase measurements before the onset of cell death.

### 2.4. Generation of Nonpolar Gene Deletions

To obtain nonpolar deletions, resistance cassettes were removed using flp-recombinase, according to the previously described protocol [24].

### 2.5. Outer Membrane Preparations

Outer membrane preparations were performed as described [25]. Outer membrane proteins were separated using 12% SDS-PAGE containing 4.8 M urea and stained with Coomassie brilliant blue [26].

### 2.6. Semiquantitative RT-PCR

To compare *malT* transcript levels, cells were grown under the indicated conditions to an OD of 1. RNA was harvested using the RNeasy Mini kit (Quiagen). DNA contamination was removed by treatment with 5 U of RQ1 RNase-free DNase (Promega) in 1x RQ1 DNase buffer for 1 h at 37°C, followed by phenol-chloroform extraction and ethanol precipitation. The subsequent reverse transcription reaction was performed using the RevertAid First Strand cDNA Synthesis Kit (Fermentas). To exclude DNA contamination, we performed a mock cDNA reaction lacking reverse transcriptase. The resulting cDNA was diluted and PCR amplified in a reaction mixture containing 2 *μ*L cDNA product, 1x PCR buffer, 0.2 *μ*M dNTPs, 4 mM MgCl_2_, 0.5 *μ*M *malT*-specific primer malTfor2 (5′-ACTCAGCCCATAAGTCGGC-3′), 0.5 *μ*M *malT*-specific primer malTrev2 (5′-CAAGACTTCAATCCCGCTAG-3′), and 1 U Taq polymerase in a total volume of 25 *μ*L. Amplification conditions were 95°C for 30 sec, 54°C for 30 sec, and 72°C for 60 sec (30 cycles), followed by 72°C for 5 min. The PCR products were subsequently analyzed on a 1% agarose gel.

## 3. Results

### 3.1. Metabolism of Glycolytic Carbon Sources Promotes Viability

We previously reported that the *ompR malT* ^con^ double mutant (strain AJW3098, Table 1) exhibits a synthetic lethal phenotype caused by the increased expression of LamB [1].

Glucose catabolite represses *mal* transcription, and thus reduces LamB expression [15, 16]. Therefore, it should not be surprising that exposure to glucose promoted survival of the *ompR malT* ^con^ mutant [1]. In contrast, the effect of maltose on *mal* gene expression can vary. Depending on expression or activity levels of the MalT protein, maltose can either enhance or inhibit MalT regulon expression [6, 27–29]. 

**Figure 2:**
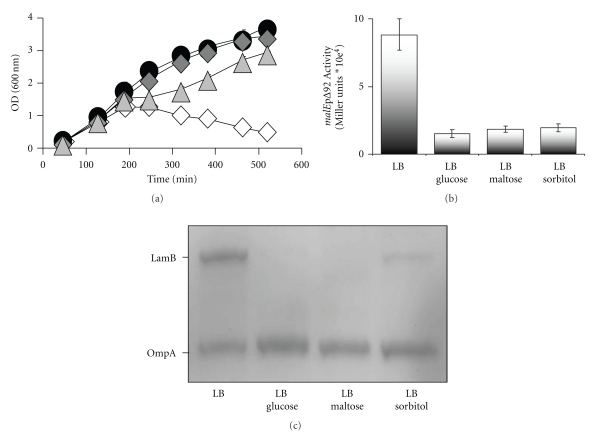
Effect of carbon sources on viability, *mal* gene transcription and LamB expression. (a) Growth curves of *ompR malT* ^con^ mutants (strain AJW3098) grown in LB at 37°C without sugars (white diamonds) or supplemented with 22 mM glucose (black circles), maltose (medium gray diamonds), or sorbitol (light gray triangles). Values represent the mean of triplicates. Error bars are only shown when greater than the symbol. (b) Effect of glycolytic carbon sources on* mal* gene transcription. *β*-galactoside activity was determined in *ompR malT* ^con^ mutants (strain AJW3098) carrying a *malE*pΔ92-*lac* reporter fusion. Cells were grown in LB without carbon source or LB supplemented with 22 mM glucose, maltose, or sorbitol. Cells were harvested at an OD_600_ of 1. Values represent the mean of triplicates. (c) Effect of glycolytic carbon sources on LamB expression. Addition of carbon sources reduces LamB levels in *ompR malT* ^con^ mutants as determined by outer membrane preparations. Cells were grown in LB at 37°C and harvested during late exponential phase. Gels were stained with Coomassie brilliant blue. Lane 1, LB no additional carbon source (LB); lane 2, LB 22 mM glucose (LB glucose); lane 3, LB 22 mM maltose (LB maltose); lane 4, LB 22 mM sorbitol (LB sorbitol).

Here, we tested if exposure to maltose enhances or suppresses lethality of the *ompR malT* ^con^mutant by growing it in LB at 37°C in the presence or absence of maltose and found that exposure to maltose suppressed death (Figure 2(a)). To test if this behavior is a general characteristic of *malT* ^con^ alleles, we tested if other *malT* ^con^ alleles behaved similarly. Several *ompR malT* ^con^ double mutants harboring a set of representative *malT* ^con^ alleles (strains AJW3732-AJW3741) [1, 11] were grown in the presence or absence of maltose. As reported previously [1], each of these double mutants died in the absence of maltose. In contrast, they all survived in its presence (data not shown). We conclude that maltose can promote viability and that this behavior is a general characteristic of *malT* ^con^ alleles.

Our finding that all the *ompR malT* ^con^ double mutants survived when exposed to maltose, combined with our previous report that disruption of *lamB* permits survival [1], supports the earlier observation that maltose can reduce *mal* gene expression in cells carrying *malT* ^con^ alleles [29]. Thus, we asked if exposure to maltose reduces MalT regulon transcription. First, we monitored MalT regulon expression of an *ompR malT* ^con^ double mutant carrying a transcriptional *malE*pΔ92*-lac* fusion [30]. We, then, directly monitored LamB expression using outer membrane preparations. Exposure of the *ompR malT* ^con^ double mutant to maltose resulted in repressed *malE*pΔ92*-lac* transcription (Figure 2(b)) and reduced LamB expression (Figure 2(c) and Supplemental Figure 1(a) which is available at doi:10.1155/2011/107023). Similarly, the *malT* ^con^ single mutant displayed reduced LamB expression in the presence of maltose (Supplemental Figure 1(b)), indicating that this effect is independent of OmpR. In contrast, WT cells and the *ompR* single mutant showed an increase in LamB expression when maltose was present (Supplemental Figure 2). We conclude that maltose supports survival of the *ompR malT* ^con^ double mutant by downregulating LamB expression.

**Figure 3:**
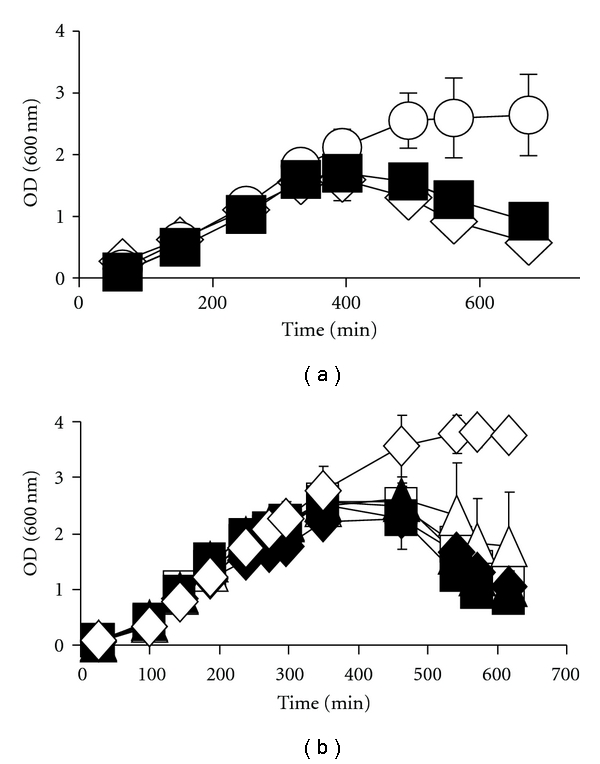
Effect of nonglycolytic carbon sources and sugar metabolism on viability. (a) Growth curves of *ompR malT* ^con^ mutants (strain AJW3098) grown in LB at 37°C without additional carbon source (white diamonds) or supplemented with serine (white circles) or succinate (black squares). Values represent the mean of triplicates. Error bars are only shown when greater than the symbol. (b) Growth curves of *ompR malT* ^con^, (strain AJW3098, diamonds), *ompR malT* ^con^ *srlA *(strain AJW3926, squares) and* ompR malT* ^con^ *srlD *(strain AJW3927, triangles) mutants grown in LB at 37°C. Black symbols, cells grown in LB without sorbitol; white symbols, cells grown in LB supplemented with 22 mM sorbitol. Values represent the mean of triplicates. Error bars are only shown when greater than the symbol.

The observation that maltose, a noncatabolite-repressing sugar, could reduce *mal* gene expression prompted us to ask if other noncatabolite-repressing sugars exert the same effect. We, therefore, grew the *ompR malT* ^con^ mutant in LB at 37°C in the presence or absence of diverse carbon sources. As expected, strong catabolite-repressing sugars (i.e., glucose, fructose, and mannitol) enabled survival (Figure 2(a) and Supplemental Table 1), whilst many noncatabolite-repressing carbon sources did not (Figure 3(a) and Supplemental Table 1). Surprisingly, some weaker or noncatabolite repressing carbon sources (i.e., sorbitol, serine, pyruvate, and mannose) promoted cell survival (Figures 2(a) and 3(a) and Supplemental Table 1). All the survival-supporting carbon sources are metabolized via glycolysis, whilst all the nonsurvival supporting carbon sources are not. Thus, some glycolysis-associated mechanism, in addition to catabolite repression, must be able to promote cell survival.

To determine why carbon sources like sorbitol promoted cell survival, we first tested if sorbitol exerts its effect by influencing *mal* gene expression. We monitored both *malE*pΔ92 transcription and LamB expression in the *ompR malT* ^con^ double mutant supplemented with sorbitol and found them to be reduced (Figures 2(b) and 2(c)). We conclude that sorbitol promotes cell survival by reducing LamB expression. 

Since sorbitol requires a transport mechanism that is different from maltose or glucose, [7, 8, 31], we asked whether survival requires transport or metabolism of the sugar. We, therefore, constructed an *ompR malT* ^con^ *srlD* triple mutant (strain AJW3927), which can transport sorbitol but not metabolize it. We also constructed an *ompR malT* ^con^ *srlA* triple mutant (strain AJW3926), which can neither transport nor metabolize sorbitol due to a polar effect of the *srlA* deletion on *srlD*. Exposure to sorbitol did not permit survival of either mutant (Figure 3(b)). These results support the argument that the effect of sorbitol on the viability of the *ompR malT* ^con^ double mutant requires metabolism of the sugar. 

### 3.2. Sorbitol Promotes Survival through a Novel Mechanism

The mechanism by which sorbitol or a metabolite exerts its effect could require either the known regulators of the maltose regulon (Figure 1), or the outer membrane porin PhoE, which has been shown to promote viability when expressed at high levels [18]. Our aim was to test whether any of these factors are required for sorbitol-promoted survival. If none of these regulators is involved, we reasoned that sorbitol must operate through a currently unknown mechanism.

In a previous report, we found that the increased expression of the PhoB regulon member PhoE, an outer membrane porin, can promote viability of the *ompR malT* ^con^ double mutant [18]. Since sugar metabolism is known to de-repress the PhoB regulon [32], we tested whether sorbitol exerts its effect by increasing PhoE abundance in the outer membrane. However, the *ompR malT* ^con^ *pstC phoE* mutant (strain AJW4197), which dies in LB [18], survived when we supplemented LB with sorbitol (data not shown). We conclude that sorbitol acts independently of PhoE.

To exert its effect on viability, products of sorbitol metabolism could act through a variety of regulators known to control *malT* expression or activity (Figure 1). For example, Mlc and CRP-cAMP affect *malT* transcription [15–17], H-NS and StpA stimulate MalT translation [13, 14], and the binding of maltotriose, MalK, MalY, Aes, or glucokinase modulates MalT activity [6, 7, 9–12, 28]. 

**Figure 4:**
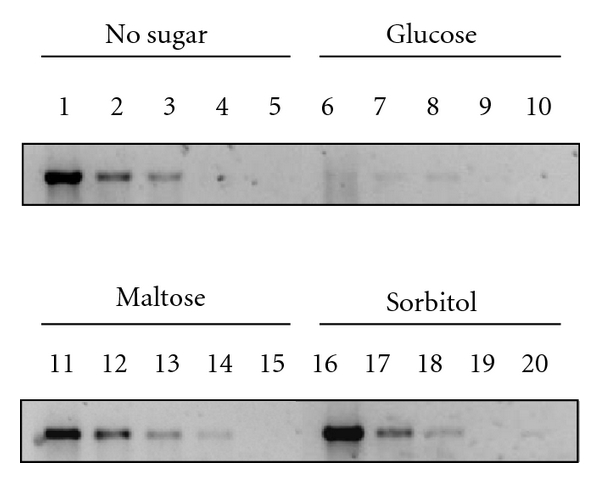
Effect of carbon sources on *malT* ^con^ transcription. Semiquantitative RT-PCR of *malT* ^con^ in the *ompR malT* ^con^ double mutant (strain AJW3098) grown under nonpermissive conditions in the absence (lane 1–5) or presence of glucose (lane 6–10), maltose (lane 11–15), or sorbitol (lane 16–20). PCR amplification was carried out with a dilution series of the cDNA: undiluted (lane 1, 6, 11, and 16), 1 : 10 dilution (lane 2, 7, 12, 17), 1 : 25 dilution (lane 3, 8, 13, and 18), 1 : 125 dilution (lane 4, 9, 14, 19), and mock control (lane 5, 10, 15, and 20).

The death of the *ompR malT* ^con^ *mlc *triple mutant (strain AJW3936) in the absence of sorbitol and its survival when sorbitol was present (Supplemental Figure 3(a)) shows that sorbitol does not exert its effect by activating the transcriptional repressor Mlc (Figure 1) [17] or by increasing its concentration and thereby repressing *malT *transcription. That sorbitol did not influence *malT *transcription was confirmed by semiquantitative RT-PCR, which showed that exposure to sorbitol did not reduce *malT* ^con^ mRNA (Figure 4). Exposure to maltose also did not reduce *malT* ^con^ mRNA, confirming earlier reports that maltose affects MalT^con^activity rather than affecting *malT* ^con^ transcription [6, 28]. Glucose, on the other hand, caused a reduction of *malT* ^con^ mRNA (Figure 4), which can be explained by its catabolite-repressing effect on *malT* transcription [15, 16]. Thus, sorbitol permits viability of the *ompR malT* ^con^ double mutant by a mechanism that does not involve altered transcription of *malT* ^con^.

Since sorbitol does not affect *malT* ^con^ transcription, we asked if it exerts its effect through any of the regulators that affect MalT^con^ activity. StpA is reported to exert a weak, activating effect on MalT regulon expression by modulating MalT translation [13]. Accordingly, we found that deletion of *stpA* in the *ompR malT* ^con^ mutant (strain AJW4028) did not promote survival (Supplemental Figure 3(b)). We further determined that sorbitol did not exert its effect through StpA (Supplemental Figure 3(b)). Since deletion of *hns* in the *ompR malT* ^con^ double mutant promoted viability [18], we could not determine whether sorbitol exerts its effect through H-NS.

**Figure 5:**
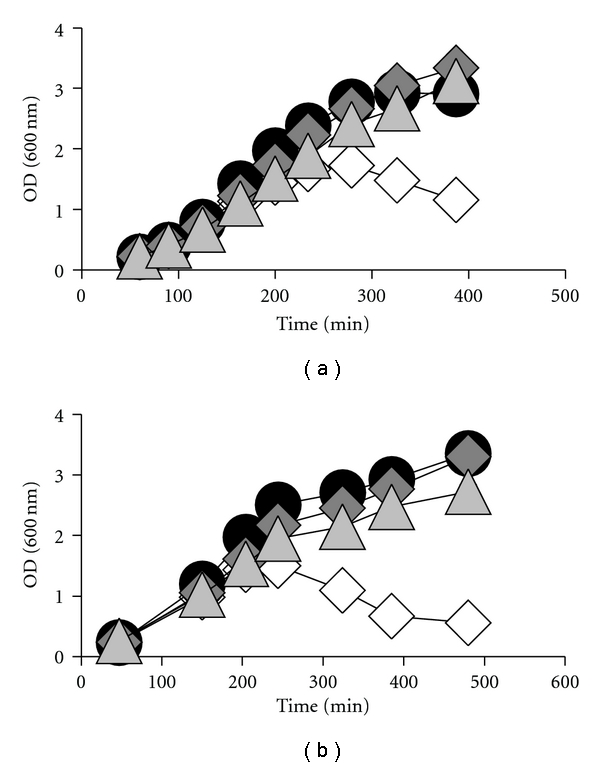
Effect of maltotriose on viability. (a) Growth curves of the *ompR malT* ^con^ *glgP* mutant (strain AJW3902) grown in LB at 37°C without sugars (white diamonds) or supplemented with glucose (black circles), maltose (dark gray diamonds), or sorbitol (light gray triangles). Values represent the mean of triplicates. Error bars are only shown when greater than the symbol. (b) Growth curves of the *ompR malT* ^con^ *malZ* mutant (strain AJW3888) grown in LB at 37°C without sugars (white diamonds) or supplemented with glucose (black circles), maltose (dark gray diamonds), or sorbitol (light gray triangles). Values represent the mean of triplicates. Error bars are only shown when greater than the symbol.

Next, we tested if sorbitol could affect MalT^con^ activity by altering maltotriose levels. We presumed that the excess carbon might be converted to glycogen and that the subsequent degradation of that glycogen might increase the intracellular maltotriose concentration, and cause endogenous induction of MalT (Figure 1) [33, 34]. Whilst glycogen phosphorylase (GlgP) is instrumental in the production of maltotriose from glycogen, maltodextrin glucosidase (MalZ) has been reported to remove maltotriose by hydrolyzing it to maltose and glucose [34]. Deletion of either *malZ* or *glgP* in the *ompR malT* ^con^ background (strains AJW3888 and AJW3902, respectively) did not rescue viability and did not diminish the ability of sorbitol to support growth (Figure 5). We conclude that the *ompR malT* ^con^ mutant is largely insensitive to maltotriose and that sorbitol likely does not suppress lethality by altering maltotriose concentrations.

MalY, Aes, MalK, and glucokinase inhibit MalT activity (Figure 1) [9–12]. We, therefore, constructed the triple mutants *ompR malT* ^con^ *malY* (strain AJW3943) and *ompR malT* ^con^ *aes* (strain AJW3947), and an *ompR malT* ^con^ *malK* triple mutant carrying a nonpolar *malK* allele (strain AJW3967) to avoid disruption of LamB expression. We further constructed an *ompR malT* ^con^ *malK glk* quadruple mutant (strain AJW4286). We monitored growth of the first two mutants under non-permissive conditions in the presence or absence of glucose, maltose, or sorbitol. Since null mutations of *malK* and/or *glk *cause defects in the importation or metabolism of maltose, mutants carrying these alleles were only grown in the presence of glucose or sorbitol. In response to all tested sugars, the mutants survived (Supplemental Figures 4(a)–4(d)). Thus, none of the sugars, including sorbitol, act through MalY, Aes, MalK, or glucokinase.

Since sorbitol-dependent survival of the *ompR malT* ^con^ mutant depends on none of the known regulatory mechanisms, we hypothesize that a novel regulatory mechanism exists, which involves posttranscriptional modulation of MalT activity. 

## 4. Discussion

### 4.1. Glycolysis Provides a New Layer of Regulation to the Maltose System

A highly complex network integrates numerous diverse signals to precisely regulate the expression and function of the maltose transport and metabolism system [6, 7, 35] (Figure 1). We now hypothesize that an additional regulatory layer exists that involves glycolysis. We base this hypothesis on the observation that the synthetic lethality of the *ompR malT* ^con^ mutant can be inhibited by growth in the presence of several glycolysis-associated carbon sources (Figures 2(a) and 3(a) and Supplemental Table 1). That a sugar like glucose or fructose can inhibit lethality is easily explained by its capacity to catabolite-repress *malTp* and *malKp* transcription and, hence, limit LamB expression [15, 16]. That sugars like maltose and sorbitol also can inhibit lethality, however, is both surprising and telling.

### 4.2. Inhibition by Maltose

Depending on MalT levels or MalT activity, the effect of maltose on MalT regulon expression can vary. In cells expressing WT MalT in large excess, it is reported that exposure to maltose causes a slight reduction (~2-fold) in *mal* gene expression [29]. However, in cells moderately overexpressing WT MalT, exposure to maltose has been reported to induce MalT regulon expression [29]. The same is true when cells express WT MalT from the endogenous gene [6, 28]. We confirmed this observation by showing that LamB levels increase in WT cells and *ompR* mutants when maltose is present (Supplemental Figure 2). If the same were true of cells that carry the *malT* ^con^ allele, then the resulting increase in LamB levels would be expected to lead to an even more premature death of the *ompR malT* ^con^ double mutant. Instead, maltose reduced *malE* transcription and LamB expression, and thus permitted survival (Figures 2(b) and 2(c) and Supplemental Figure 1). 

In cells carrying *malT* ^con^ alleles, exposure to maltose has been reported to induce expression at the *malE* promoter, with the notable exception of highly constitutive MalT^con^ proteins [27, 29]. In contrast, we found that exposure to maltose causes reduced *malE* promoter activity in the *ompR malT* ^con^ double mutant (Figure 2(b)) and reduced LamB levels in both the *malT* ^con^ single and *ompR malT* ^con^ double mutants (Figure 2(c) and Supplemental Figure 1), permitting the *ompR malT* ^con^ double mutant to survive. Since survival was observed in all 10 *ompR malT* ^con^ mutants tested, representing each location cluster and inhibition class [11], it is likely that the observed inhibitory response is a general characteristic of MalT^con^ proteins. The discrepancy between this and the previous reports could be due to utilization of different strain backgrounds (MC4100 versus AJW678) or of different media (minimal medium with glycerol as the base carbon source and supplemented with maltose versus LB supplemented with maltose).

In a previous study, exposure to maltose in the context of high expression of MalT^WT^ caused a moderate 2-fold reduction of *malE *transcription [29]. To explain this result, a model was proposed in which overproduction of MalT^WT^ results in a large number of MalT^WT^ oligomers that substitute for CRP. This would result in formation at the *malEp* and *malKp* promoters of a less active nucleoprotein complex containing only MalT [36]. A further development of this model proposed unlimited aggregation of MalT to be responsible for the inhibition of *malEp* transcription at high concentrations of MalT [37]. For these models to explain our observations, the native gene would have to express enough MalT^con^ to permit successful competition with CRP for DNA binding. Furthermore, those constitutively active proteins would have to become more active in response to maltose. Finally, the combination would have to be able to exert a 2-fold larger effect (4-fold inhibition) than did the wild-type protein expressed from a multicopy plasmid (2-fold inhibition). We think it is more likely that maltose acts upon MalT^con^ in a manner similar to that of sorbitol.

### 4.3. Inhibition by Sorbitol

Sorbitol has never been reported to influence the maltose system or inhibit MalT regulon transcription; thus, the inhibitory mechanism through which it operates must be novel. With the notable exceptions of H-NS and the CRP-cAMP complex, we tested the involvement of each known MalT regulator (Figure 1) and found that none are required (Figures 4 and 5 and Supplemental Figures 3 and 4). We ruled out the CRP-cAMP complex, because sorbitol causes only weak catabolite repression [8, 38]. This argument is further supported by the observation that exposure to sorbitol did not alter *malT* ^con^ mRNA levels (Figure 4). We also excluded H-NS, since, to our knowledge, the global regulator has never been reported to respond to glycolysis. Thus, we hypothesize that sorbitol exerts its effect on *mal* gene transcription through a novel mechanism that is independent of the currently reported regulators and signals. 

Our studies show that the lethal phentoype caused by the MalT^con^ protein used in this study is insensitive to both the inducer maltotriose (Figure 5) and the inhibitor MalK [1, 39] (data not shown). Although MalK can exert a small effect on the activity of this MalT^con^ protein, an observation made when we tested media that does not contain the MalT inducer trehalose, this small effect was insufficient to influence the lethal phenotype (Reimann and Wolfe, unpublished data). Thus, stripped of the two primary layers of regulation provided by maltotriose and MalK, the *ompR* *malT* ^con^ double mutant exposes an otherwise undetectable layer of regulation. That sorbitol must be metabolized to inhibit MalT regulon transcription (Figure 3(b)) suggests the existence of a central metabolite that modulates MalT regulon expression. 

The identity of this central metabolite remains unknown. However, recent reports that CRP and other transcription factors can become acetylated [40–42] coupled with the knowledge that the protein deacetylase CobB depends on NAD^+^ for its function [43] raises the exciting possibility that increased glycolytic flux due to metabolism of the excess sorbitol results in acetylation of CRP, MalT, or some other component of the nucleoprotein complex that modulates *malE *and *malK* transcription, resulting in inhibition and thus survival. 

The concept of a glycolytic metabolite opens up the possibility that maltose, glucose, fructose, and other glycolytic carbon sources could work through the same mechanism. In the case of glucose and fructose, however, the effect is normally concealed by their strong catabolite-repressing effect. Likewise, in cells harboring a MalT^WT^ protein, the strong regulatory effects of maltotriose and MalK would normally counterbalance the regulatory effect of maltose metabolism.

## Supplementary Material

The supplementary material include:
Table S1, Survival of the ompR malT^con^ double mutant in the presence of various sources
Figure S1, effect of carbon sources on LamB expression
Figure S2, effect of maltose on LamB expression

## Figures and Tables

**Table 1:** Strains, plasmids, and reporter fusions used in this study.

	Relevant genotype	Reference
AJW678	*thi-1thr-1*(Am) *leuB6 metF159*(Am) *rpsL136 lacX74 *	[20]
AJW2050	AJW678 *ompR*::Tn*10 *	[44]
AJW3098	AJW678 *ompR*::Tn*10 * *malT* ^con^ *T949A *	[1]
AJW3499	AJW678 *malT* ^con^ *T949A *	[1]
AJW3732	AJW678 *ompR*::Tn*10 * *malT* ^con^ (S358I)	[1]
AJW3733	AJW678 *ompR*::Tn*10 * *malT* ^con^ (W317P)	[1]
AJW3734	AJW678 *ompR*::Tn*10 * *malT* ^con^ (R242C)	[1]
AJW3735	AJW678 *ompR*::Tn*10 * *malT* ^con^ (A244E)	[1]
AJW3736	AJW678 *ompR*::Tn*10 * *malT* ^con^ (A236S)	[1]
AJW3737	AJW678 *ompR*::Tn*10 * *malT* ^con^ (A236D)	[1]
AJW3738	AJW678 *ompR*::Tn*10 * *malT* ^con^ (P10Q)	[1]
AJW3739	AJW678 *ompR*::Tn*10 * *malT* ^con^ (R242S)	[1]
AJW3740	AJW678 *ompR*::Tn*10 * *malT* ^con^ (T38R)	[1]
AJW3741	AJW678 *ompR*::Tn*10 * *malT* ^con^ (S5L)	[1]
AJW3888	AJW678 *ompR*::Tn*10 * *malT* ^con^ *T949A ΔmalZ*::Km	This study
AJW3902	AJW678 *ompR*::Tn*10 * *malT* ^con^ *T949A ΔglgP*::Km	This study
AJW3926	AJW678 *ompR*::Tn*10 * *malT* ^con^ *T949A ΔsrlA*::Km	This study
AJW3927	AJW678 *ompR*::Tn*10 * *malT* ^con^ *T949A ΔsrlD*::Km	This study
AJW3936	AJW678 *ompR*::Tn*10 * *malT* ^con^ *T949A Δmlc*::Km	This study
AJW3943	AJW678 *ompR*::Tn*10 * *malT* ^con^ *T949A ΔmalY*::Km	This study
AJW3947	AJW678 *ompR*::Tn*10 * *malT* ^con^ *T949A Δaes*::Km	This study
AJW3967	AJW678 *ompR*::Tn*10 * *malT* ^con^ *T949A ΔmalK*::frt	This study
AJW4023	AJW678 *ompR*::Tn*10 * *malT* ^con^ *T949A Δhns*::Km	This study
AJW4028	AJW678 *ompR*::Tn*10 * *malT* ^con^ *T949A ΔstpA:*:Km	This study
AJW4197	AJW678 *ompR*::Tn*10 * *malT* ^con^ *T949A pstC*::frt *phoE*::km	This study
AJW4286	AJW678 *ompR*::Tn*10 * *malT* ^con^ *T949A ΔmalK*::frt ∆*glk*::Km	This study

Reporter fusions		
*malE*pΔ92-*lac *	*trp*::[KanR-*malE*pΔ92-*lac*]_op_	[30]
