# Supplementary material for: Exposure to Glycolytic Carbon Sources Reveals a Novel Layer of Regulation for the MalT Regulon
Source: Int J Microbiol. 2011 Aug 14;2011:107023. doi: 10.1155/2011/107023 (PMC3161199; doi:10.1155/2011/107023)
Supplement: Supplementary file 1 — The supplementary material include: Table S1, Survival of the ompR malTcon double mutant in the presence of various sources Figure S1, effect of carbon sources on LamB expression Figure S2, effect of maltose on LamB expression [file 107023.f1.pdf]

Supplemental Table S1: Survival of the *ompR malT*^{con} double mutant in the presence of various carbon sources. The relative catabolite repressing strength and glycolysis association of the carbon sources as well as the resulting death phenotype are indicated with “+” and ”-“.

Carbon source	Catabolite-repression	glycolytic	survival
Glucose	+++	+++	+++
Fructose	+++	+++	+++
Mannitol	+++	+++	+++
Glucuronate	+++	+++	+++
Pyruvate	++	+++	+++
Sorbitol	+	+++	++
Mannose	+	+++	+++
Serine	-	+++	+++
Maltose	-	+++	+++
Succinate	-	-	-
Aspartate	-	-	-
Tryptophan	-	-	-
Glutamate	-	-	-
Glycine	-	-	-
Proline	-	-	-
Betaine	-	-	-

Supplemental Figure Legends

Supplemental Figure 1. Effect of carbon sources on LamB expression.

Addition of carbon sources reduces LamB levels in (a) *ompR malT*^{con} and (b) *malT*^{con} mutants as determined by outer membrane preparations. Cells were grown in LB at 37 °C and harvested during late exponential phase. Gels were stained with Coomassie brilliant blue. Lane 1, LB no carbon source (LB); lane 2, LB 22 mM maltose (LB mal); lane 3, LB 22 mM glucose (LB gluc); lane 4, LB 22 mM sorbitol (LB sor).

Supplemental Figure 2. Effect of maltose on LamB expression.

Addition of maltose increases LamB levels in WT cells (lane 1 & 2) and the *ompR* mutant (lane 3 & 4) as determined by outer membrane preparations. Cells were grown in LB at 37 °C and harvested during late exponential phase. Gels were stained with Coomassie brilliant blue. Lane 1, WT cells no carbon source (LB); lane 2, WT cells 22 mM maltose (LB mal); lane 3, *ompR* mutant no carbon source (LB); lane 4, *ompR* mutant 22 mM maltose (LB mal).

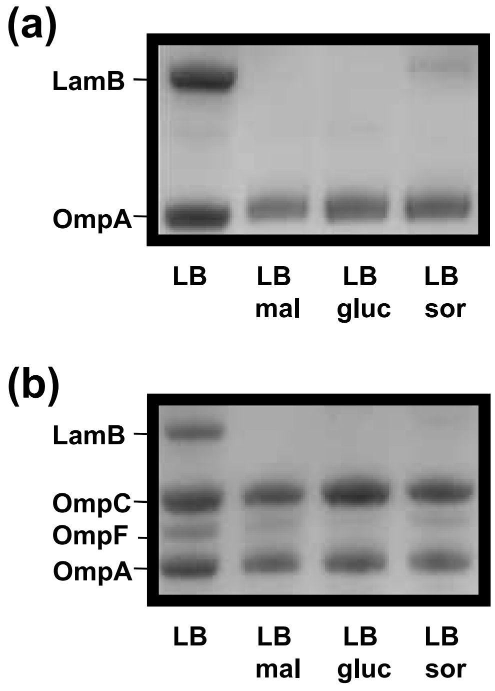

Reimann & Wolfe Supplemental Figure 1

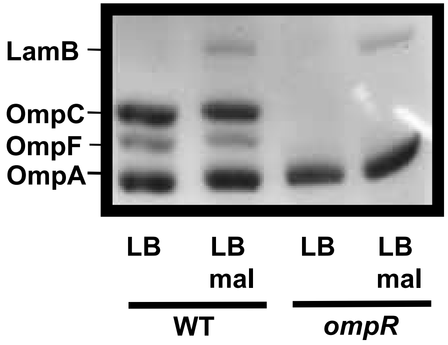

Reimann & Wolfe Supplemental Figure 2
