# Supplementary material for: Exposure to Glycolytic Carbon Sources Reveals a Novel Layer of Regulation for the MalT Regulon
Source: Int J Microbiol. 2011 Aug 14;2011:107023. doi: 10.1155/2011/107023 (PMC3161199; doi:10.1155/2011/107023)
Supplement: Supplementary file 2 [file 107023.f2.pdf]

Supplemental Table S1: Survival of the *ompR malT*^{con} double mutant in the presence of various carbon sources. The relative catabolite repressing strength and glycolysis association of the carbon sources as well as the resulting death phenotype are indicated with “+” and ”-“.

Carbon source	Catabolite-repression	glycolytic	survival
Glucose	+++	+++	+++
Fructose	+++	+++	+++
Mannitol	+++	+++	+++
Glucuronate	+++	+++	+++
Pyruvate	++	+++	+++
Sorbitol	+	+++	++
Mannose	+	+++	+++
Serine	-	+++	+++
Maltose	-	+++	+++
Succinate	-	-	-
Aspartate	-	-	-
Tryptophan	-	-	-
Glutamate	-	-	-
Glycine	-	-	-
Proline	-	-	-
Betaine	-	-	-

Supplemental Figure Legends

Supplemental Figure 1. Effect of carbon sources on LamB expression. Addition of carbon sources reduces LamB levels in (a) *ompR* $malT^{con}$ and (b) $malT^{con}$ mutants as determined by outer membrane preparations. Cells were grown in LB at 37 °C and harvested during late exponential phase. Gels were stained with Coomassie brilliant blue. Lane 1, LB no carbon source (LB); lane 2, LB 22 mM maltose (LB mal); lane 3, LB 22 mM glucose (LB gluc); lane 4, LB 22 mM sorbitol (LB sor).

Supplemental Figure 2. Effect of maltose on LamB expression. Addition of maltose increases LamB levels in WT cells (lane 1 & 2) and the *ompR* mutant (lane 3 & 4) as determined by outer membrane preparations. Cells were grown in LB at 37 °C and harvested during late exponential phase. Gels were stained with Coomassie brilliant blue. Lane 1, WT cells no carbon source (LB); lane 2, WT cells 22 mM maltose (LB mal); lane 3, *ompR* mutant no carbon source (LB); lane 4, *ompR* mutant 22 mM maltose (LB mal).

Supplemental Figure 3. Carbon sources do not act through the regulators Mlc or StpA. Growth curves of the triple mutants *ompR* $malT^{con}$*mlc* (strain AJW3936) (a) and *ompR* $malT^{con}$*stpA* (strain AJW4028) (b) grown in LB at 37 °C without sugars (white diamonds) or supplemented with 22 mM glucose (black circles), maltose (medium gray diamonds), or

sorbitol (light gray triangles). Values represent the mean of triplicates. Error bars are only shown when greater than the symbol.

Supplemental Figure 4. Carbon sources do not act through the inhibitors MalK, MalY, Aes, or glucokinase. Growth curves of the triple mutants *ompR* $malT^{con}$*malK* (strain AJW3967) (a), *ompR* $malT^{con}$*malY* mutants (strain AJW3943) (b) , *ompR* $malT^{con}$*aes* (strain AJW3947) (c), or the quadruple mutant *ompR* $malT^{con}$*malK glk* (strain AJW4286) (d) grown in LB at 37 °C without sugars (white diamonds) or supplemented with 22 mM glucose (black circles), maltose (medium gray diamonds), or sorbitol (light gray triangles). Values represent the mean of triplicates. Error bars are only shown when greater than the symbol. Since *glk* and *malK* mutants cannot metabolize/import maltose, these mutants were only grown in the presence of glucose or sorbitol.

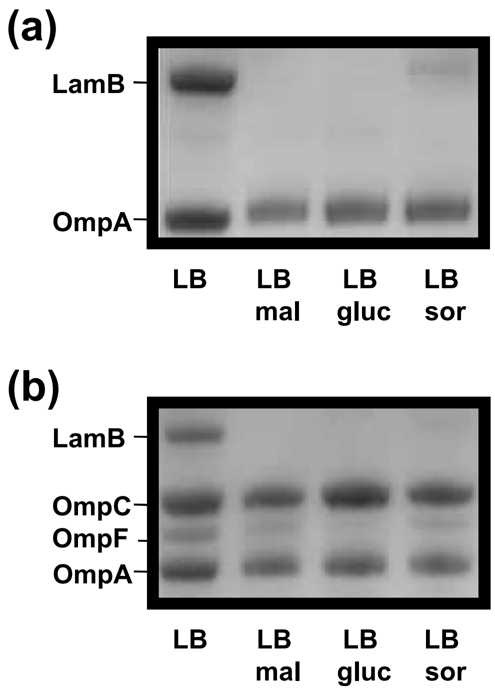

Reimann & Wolfe Supplemental Figure 1

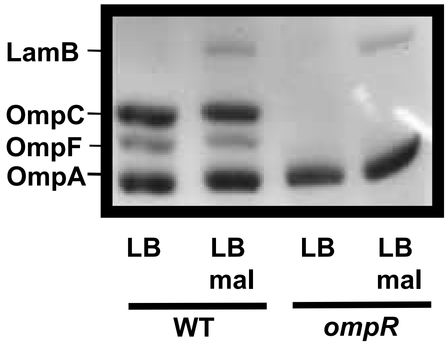

Reimann & Wolfe Supplemental Figure 2

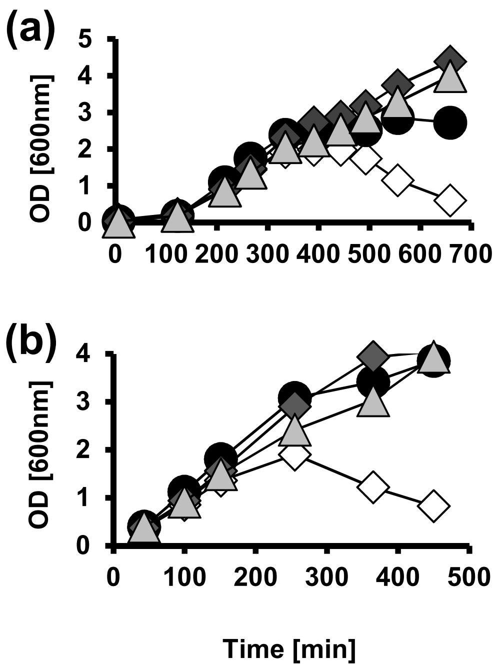

Reimann & Wolfe Supplemental Figure 3

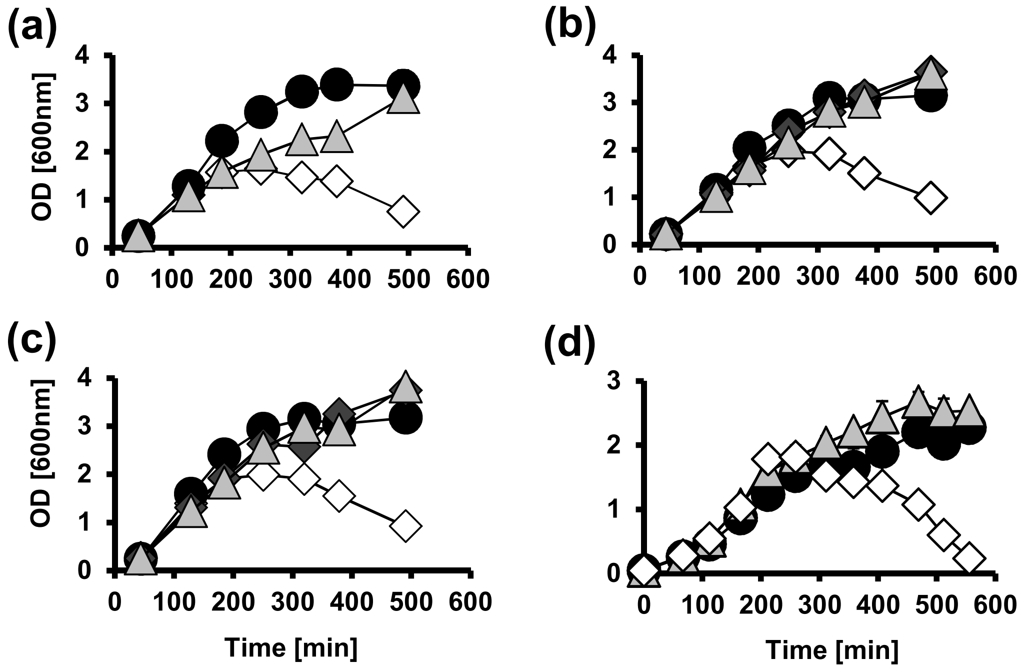

Reimann & Wolfe Supplemental Figure 4
